# Experimental infections in red-legged partridges reveal differences in host competence between West Nile and Usutu virus strains from Southern Spain

**DOI:** 10.3389/fcimb.2023.1163467

**Published:** 2023-06-15

**Authors:** Francisco Llorente, Rafael Gutiérrez-López, Elisa Pérez-Ramirez, María Paz Sánchez-Seco, Laura Herrero, Miguel Ángel Jiménez-Clavero, Ana Vázquez

**Affiliations:** ^1^ Centro de Investigación en Sanidad Animal (CISA-INIA), Consejo Superior de Investigaciones Científicas (CSIC), Valdeolmos, Madrid, Spain; ^2^ Centro Nacional de Microbiología, Instituto de Salud Carlos III, Majadahonda, Madrid, Spain; ^3^ CIBER Enfermedades Infecciosas (CIBERINFEC), Madrid, Spain; ^4^ CIBER Epidemiología y Salud Pública (CIBERESP), Madrid, Spain

**Keywords:** vector-borne diseases, arbovirus, flavivirus, birds, experimental infection

## Abstract

**Introduction:**

West Nile virus (WNV) and Usutu virus (USUV) are emerging zoonotic arboviruses sharing the same life cycle with mosquitoes as vectors and wild birds as reservoir hosts. The main objective of this study was to characterize the pathogenicity and course of infection of two viral strains (WNV/08 and USUV/09) co-circulating in Southern Spain in a natural host, the red-legged partridge (*Alectoris rufa*), and to compare the results with those obtained with the reference strain WNV/NY99.

**Methods:**

WNV inoculated birds were monitored for clinical and analytical parameters (viral load, viremia, and antibodies) for 15 days post-inoculation.

**Results and discussion:**

Partridges inoculated with WNV/NY99 and WNV/08 strains showed clinical signs such as weight loss, ruffled feathers, and lethargy, which were not observed in USUV/09-inoculated individuals. Although statistically significant differences in mortality were not observed, partridges inoculated with WNV strains developed significantly higher viremia and viral loads in blood than those inoculated with USUV. In addition, the viral genome was detected in organs and feathers of WNV-inoculated partridges, while it was almost undetectable in USUV-inoculated ones. These experimental results indicate that red-legged partridges are susceptible to the assayed Spanish WNV with pathogenicity similar to that observed for the prototype WNV/NY99 strain. By contrast, the USUV/09 strain was not pathogenic for this bird species and elicited extremely low viremia levels, demonstrating that red-legged partridges are not a competent host for the transmission of this USUV strain.

## Introduction

West Nile virus (WNV) and Usutu virus (USUV) are arthropod-borne viruses belonging to the family *Flaviviridae*, genus *Flavivirus*. Both viruses belong to the Japanese encephalitis virus (JEV) antigenic complex (Calisher & Gould, 2003) and have a similar ecology. Their life cycle involves multiple bird species as amplifying hosts and a wide range of mosquitoes as vectors, with *Culex pipiens* as the main species involved (Busquets et al., 2008; Vázquez et al., 2011; Calzolari et al., 2012; Fros et al., 2015). Although horses and humans are susceptible to the infection (Castillo-Olivares and Wood, 2004; Gaibani et al., 2012; Aberle et al., 2018; Guerrero-Carvajal et al., 2021), they are not competent hosts due to their low and transient viremia, insufficient to transmit the virus to feeding mosquitoes (McLean et al., 2002; Vilibic-Cavlek et al., 2020).

West Nile virus is the etiological agent of a zoonotic disease with a severe impact on human and animal health (Nikolay, 2015). This emerging pathogen has broadly expanded during the last 20 years and is nowadays considered one of the most widespread arboviruses in the world, being present in all continents except Antarctica (Jiménez-Clavero, 2012; Paz, 2015). Although infection in humans is, in most cases, asymptomatic or results in mild clinical signs, a low percentage (<1%) of infected individuals develop severe neurological disease, including encephalitis or meningoencephalitis (Nash et al., 2001; Campbell et al., 2002). In Europe, WNV has been circulating with increasing intensity in the last two decades, spreading to most countries in the continent, except the Northernmost (European Centre for Disease Prevention and Control (ECDC), 2022). Important outbreaks of WNV lineages 1 and 2 in horses and humans have occurred also recently, mainly in eastern countries (2018), Spain (2020), and Italy (2022) (Bakonyi & Haussig, 2020; European Centre for Disease Prevention and Control (ECDC), 2022; Figuerola et al., 2022). Outbreaks in wild birds have also been observed to affect species such as the Northern goshawk (*Accipiter gentilis*) (Aguilera-Sepúlveda et al., 2022), snowy owl (*Bubo scandiacus*), Chinese merganser (*Mergus squamatus*), black-tailed gull (*Larus crassirostris*), great tit (*Parus major*) (Santos et al., 2022), griffon vulture (*Gyps fulvus*), the little owl (*Athene noctua*) (Bravo-Barriga et al., 2021). Although abundant information exists regarding experimental infections of birds with WNV, especially with the WNV/NY99 strain (Komar et al., 2003; Pérez-Ramírez et al., 2014), studies about the pathogenicity of Euro-Mediterranean strains in different avian species are still scarce (Sotelo et al., 2011; Dridi et al., 2013; Lim et al., 2014; Spedicato et al., 2015). In fact, the pathogenicity of new strains is worth to be studied in order to obtain a better understanding of the eco-epidemiology of WNV.

Usutu virus has spread to Europe over the last two decades mainly leading to avian mortalities (Weissenböck et al., 2002; Clé et al., 2019), and although infections in humans are considered asymptomatic or with mild clinical signs, encephalitis or meningoencephalitis cases have also been reported (Cavrini et al., 2009; Pecorari et al., 2009; Gaibani et al., 2013; Nagy et al., 2019; Pacenti et al., 2019). So far, eight lineages of USUV have been described: five European lineages (Europe 1–5) and three African lineages (Africa 1–3) (Cadar et al., 2017). Usutu virus has been identified from mosquitos (Busquets et al., 2008; Calzolari et al., 2012; Jöst et al., 2011; Vázquez et al., 2011) and birds, such as common blackbird (*Turdus merula*) and great gray owl (*Strix nebulosa*), and specific antibodies have been found in common blackbird (*T. merula*), carrion crow (*Corvus corone*), Eurasian magpie (*Pica pica*), house sparrow (*Passer domesticus*), red-legged partridge (*Alectoris rufa*), and common turkey (*Meleagris gallopavo*), among others (Figuerola et al., 2009; Llorente et al., 2013; Clé et al., 2019; Bravo-Barriga et al., 2021; Napp et al., 2021; Marzal et al., 2022). Although USUV distribution in Europe is increasing considerably (Cadar et al., 2017), scarce data from experimental infection in birds are available (Chvala et al., 2005; Chvala et al., 2006; Benzarti et al., 2020; Escribano-Romero et al., 2021; Kuchinsky et al., 2021; Kuchinsky et al., 2022).

In nature, WNV and USUV are often found co-circulating in the same areas, probably due to their similar ecology (Llorente et al., 2013). In Spain, the circulation of WNV and USUV has been known since 2003 (Figuerola et al., 2007) and 2006, respectively (Busquets et al., 2008). West Nile virus was isolated for the first time in Spain in 2007 from vertebrate hosts (golden eagle, *Aquila chrysaetos*; Jimenez-Clavero et al., 2008) and identified in vectors in 2006 (*C. pipiens*; Vázquez et al., 2010) and 2008 (*Culex perexiguus*; Vázquez et al., 2011). Usutu virus was detected in vectors in 2006 (*C. pipiens*; Busquets et al., 2008). In addition, in the south of Spain, serological evidence supports the co-circulation of both viruses in the same populations of red-legged partridge (Llorente et al., 2013). This fact could indicate that this species can play a relevant role in the epidemiology of these flaviviruses in Spain. Moreover, it has been shown as a suitable animal model for WNV experimental research. Notably, it was found to reach viremic levels high enough to infect mosquitoes (i.e., it is a competent host). Furthermore, it is useful to discriminate between WNV strains in terms of pathogenicity (Sotelo et al., 2011; Pérez-Ramírez et al., 2018; Gamino et al., 2021). However, the susceptibility of this wild bird species to USUV infection and disease, as well as its competence for virus transmission and overall epidemiological role, is still unclear.

In Southern Spain, WNV lineage 1 and a USUV Africa 2 lineage strains were detected from mosquitoes *C. perexiguus* in 2008 and 2009, respectively (Vázquez et al., 2011). In this area, co-circulation of WNV and USUV has been detected in birds and vectors (Llorente et al., 2013), and numerous human cases of WNV infections have been noted (Rodríguez-Alarcón et al., 2021). However, the pathogenicity of these WNV and USUV strains in wild bird hosts is still unknown.

Therefore, the main aim of this study is to evaluate the course of the infection of the WNV strain (WNV/08) and the USUV strain (USUV/09) isolated from Southwestern Spain in bird hosts by means of experimental inoculations in the red-legged partridge bird model. We examined mortality, morbidity, viremia, and virus load in blood, feathers, oral swabs, and organs and seroconversion in order to evaluate the pathogenic potential of these two flaviviruses and the potential role of the red-legged partridge as a competent reservoir host in nature.

## Materials and methods

### Viruses and virus preparations

Two strains of WNV (WNV/NY99, North American reference strain, and WNV/08 Spanish strain) and one Spanish strain of USUV (USUV/09) were used in this study. The origins of the Spanish strains can be consulted in Vázquez et al. (2011), and details about passage since isolation are described in **Table 1**. All the strains were titrated by plaque assay in Vero cells, and virus titers were given in plaque-forming units (pfu).

**Table 1:** Viral strains used in this study, origins, sources, and the number of passages in cell culture.

Virus strain	Geographic origin	Species	Year of isolation	Cell passage number	GenBank accession number	Source of the strain
WNV/NY99 (NY99crow-V76/1)	New York NY (USA)	American crow	1999	VR-6p	FJ51394	Diagnostic Virology Laboratory Dept. of Agriculture (USDA), Ames, IA, USA
WNV/08 (HU6365/08)	(Spain)	*Culex perexiguus*	2008	VR-2p	JF707789	Instituto de Salud Carlos III (ISCIII)
USUV/09 (HU10279/09)	(Spain)	*C. perexiguus*	2009	VR-4p	MN813489	Instituto de Salud Carlos III (ISCIII)

VR, vero cells.

### Experimental inoculations of red-legged partridges

Six-week-old red-legged partridges (*A. rufa*) (n = 39) were obtained from the Lugar Nuevo breeding facility (Estación de Referencia de la Perdiz Roja, Consejería de Medio Ambiente y Ordenación del Territorio-Junta de Andalucía, Andújar, Spain, 38°16′N; 4°6′W). The partridges were transported to the biosafety level 3 (BSL-3) facilities at CISA-INIA (Centro de Investigación en Sanidad Animal, Valdeolmos, Spain) and distributed in four groups in wire mesh cages (three cages with 10 birds and another with 9) after external deparasitation. The partridges were provided with a commercial diet for game birds and water *ad libitum* throughout the experiment. Previous exposure of the individuals to WNV and USUV was evaluated serologically using a commercially available competitive ELISA (INgezim West Nile Compac, INGENASA, Madrid, Spain) and virologically by real-time RT-PCR (Del Amo et al., 2013).

After 7 days for acclimatization, three groups composed of 10, 10, and 9 red-legged partridges in their seventh week of age were inoculated subcutaneously in the neck (10^4^ pfu/individual) with the strains WNV/NY99, WNV/08, and USUV/09, respectively. The inocula were diluted in 100 μl/individual of phosphate-buffered saline (PBS) with 0.2% bovine serum albumin (BSA). The fourth group (negative control group, n = 10) was sham-inoculated with an equivalent volume of PBS with 0.2% BSA and maintained in a separate cage.

Animal care, handling, and experimental procedures were authorized by the INIA Committee of Ethics and Animal Experimentation (reference: 10/033826).

### Clinical follow-up and collection of samples

Partridges were observed daily for disease symptoms up to 15 days post-inoculation (dpi). All the birds were weighed at 1, 3, 5, 7, 9, 12, and 15 dpi. To assess the course of viremia and viral load in blood, samples were collected at 1, 3, 5, 7, and 9 dpi. Blood (0.1 ml) was collected in sterile polypropylene tubes filled with 0.9 ml of BA-1 diluent (Hanks M-199 salts, 0.05 M Tris, pH 7.6, 1% BSA, 0.35 g/L of sodium bicarbonate, 100 U/ml of penicillin, 100 μg/ml of streptomycin, and 1 μg/ml of amphotericin B) and stored at −80°C until analysis. To assess the antibody response, a second blood sample (0.1–0.2 ml/individual) was collected at 3, 5, 7, 9, 12, and 15 dpi in dry tubes and allowed to clot at 37°C for 1 h followed by overnight incubation at 4°C to obtain serum. Oropharyngeal swabs and immature rump feathers were collected at 1, 3, 5, 7, 9 12, and 15 dpi. Swabs were placed in sterile polypropylene tubes containing 1 ml of PBS, and feathers were collected in empty sterile polypropylene tubes. Both types of samples were stored at −80°C until analysis. At 15 dpi, three animals from each group were necropsied, and samples (approximately 0.1 g) of the brain, heart, kidney, spleen, and liver were collected in tubes containing 0.9 ml of PBS for PCR analysis. The individual that died during the experiment was necropsied, and samples of the brain, heart, kidney, spleen, and liver were collected. Single-use scalpels and forceps were used to avoid cross-contamination. Sham-inoculated (negative control group) partridges were handled, sampled, and analyzed in parallel, exactly in the same way as the virus-inoculated groups.

### Virus detection assays

Viremia was measured by standard plaque formation assays as described (Payne et al., 2006). The viral genome was extracted following different previous preparation steps depending on the type of sample. Blood samples, diluted in 0.9 ml of BA-1 (see above), were centrifuged at 6,000 ×*g* for 5 min, and 200 μl from the supernatant was used for RNA extraction. For feathers, the vascular pulp was aseptically removed from the umbilicus with forceps and placed in tubes containing 0.9 ml of PBS (one pulp per sampling day and animal). Tissues from necropsies and feather pulps in PBS were homogenized for 2 min at 30 cycles/s using a TissueLyser homogenizer (QIAGEN, Valencia, CA, USA) followed by a centrifugation step at 6,000 ×*g* for 5 min to clarify homogenates. The swab suspensions were vortexed and then clarified at 6,000 ×*g* for 5 min. RNA from blood, organs, feathers, and swabs was extracted using BioSprint 15 platform (QIAGEN) and subjected to real-time RT-PCR (RRT-PCR), as described previously (Del Amo et al., 2013). Samples with Ct > 40.0 were considered negative (Del Amo et al., 2013).

### Antibody detection assays

Virus-neutralizing antibodies in serum were titrated by virus neutralization test (VNT) in 96-well microplates as described (Llorente et al., 2019). Serum dilutions (from 1:5 to 1:1,280) were assayed in parallel against the reference strains WNV Eg-101 (GenBank accession number AF260968) or USUV SAAR‐1776 (GenBank accession number AY453412).

### Statistical analyses

The survival curves between the groups of partridges infected with the different strains were calculated by Kaplan–Meier analysis and analyzed by log-rank test using IBM SPSS (IBM Corporation). Differences in weight variations during the experiment (dependent variable) among groups were analyzed by means of linear mixed models. The variables day, viral strain, and the interaction between both were included as independent factors. Individual identity was included as a random factor. Differences in body weight variation among groups at specific dpi were analyzed by a pairwise t-test. Viral genome load in blood, feathers, and oral swabs were compared between the groups infected with WNV using the Kruskal–Wallis test. Statistical analyses were run in R software 3.2.5 (R Core Team, 2016) using the package *lme4* (Bates et al., 2015).

## Results

### Pathogenicity for red-legged partridge

**Figure 1:**
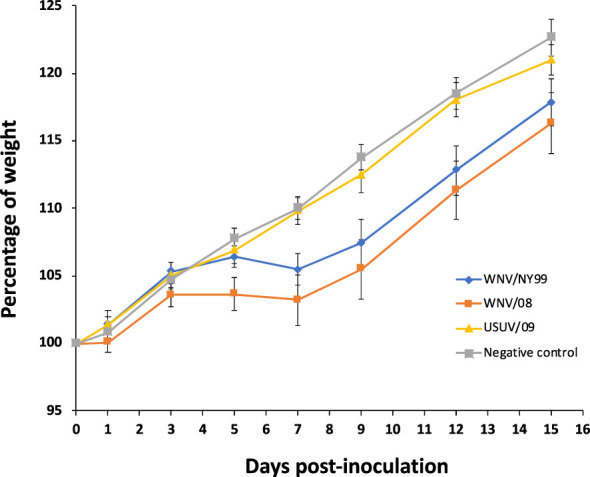
Weight curve of red-legged partridges after virus inoculation expressed as the percentage of initial weight through different days post-inoculation. Bars represent the standard error of the mean.

All partridges inoculated with either WNV/08 or WNV/NY99 showed unspecific clinical signs (e.g., weight loss, ruffled feathers, and lethargy), while no clinical signs were observed in partridges inoculated with USUV/09. Statistically significant differences in weight variation during the infection were observed between groups. Weight gain throughout the experiment was significantly lower in the individuals infected with WNV/NY99 (estimate = −0.541; Std. error = 0.089; p-value < 0.001) or with WNV/08 (estimate = −0.478; Std. error = 0.087; p-value < 0.001) in comparison with the negative control group. However, the group infected with USUV/09 did not show a significant difference in weight during the experiment in comparison with the negative control group (estimate = −0.118; Std. error = 0.089; p-value = 0.181) (**Figure 1**). Statistically significant differences in body weight variation between the control and WNV/08-inoculated group were observed at specific dpi (at 7 dpi, p = 0.02; at 9 dpi, p = 0.02; at 12 dpi, p = 0.04) and between control and WNV/NY99 (at 7 dpi, p = 0.02; at 9 dpi, p = 0.02).

Only one bird infected with WNV/08 died at 7 dpi, but non-significant differences in mortality rate in this group in comparison with the other groups were detected (p = 0.317).

Birds inoculated with WNV/NY99 or WNV/08 strains started to show symptoms at 5–7 dpi. From this day on, surviving partridges started to gain weight, although the average weight in the WNV-inoculated groups remained lower than in the USUV group and the negative control group at the end of the experiment (**Figure 1**). The control group stayed healthy and steadily increased weight during the experiment as expected for the age of the birds (7 weeks old at the start of the experiment).

### Viral detection in blood, feathers, oral swabs, and organs

**Figure 2:**
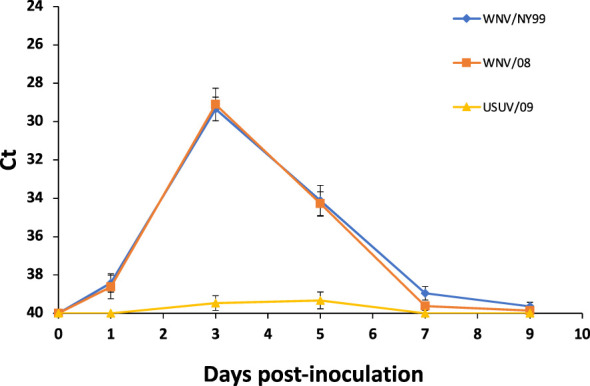
Mean daily blood viral genome load for the three inoculated virus groups. Each point represents the mean Ct obtained for the surviving individuals at different times post-inoculation. Bars represent the standard error of the mean.

All WNV-inoculated partridges developed a detectable virus genome in blood from 1 to 9 dpi, with a peak of viral RNA load at 3 dpi. Non-significant differences in average Ct values between WNV strains were found throughout the experiment (29.35 for WNV/NY99 and 29.11 for WNV/08) (χ^2 = ^0.79, d.f. = 1, p-value = 0.37) (**Figure 2**). Regarding the USUV/09-inoculated group, viral RNA load in blood was detected, at a high Ct value (i.e., >36), in 60% (6/10) of the individuals at 3 or 5 dpi (**Figure 2**).

**Figure 3:**
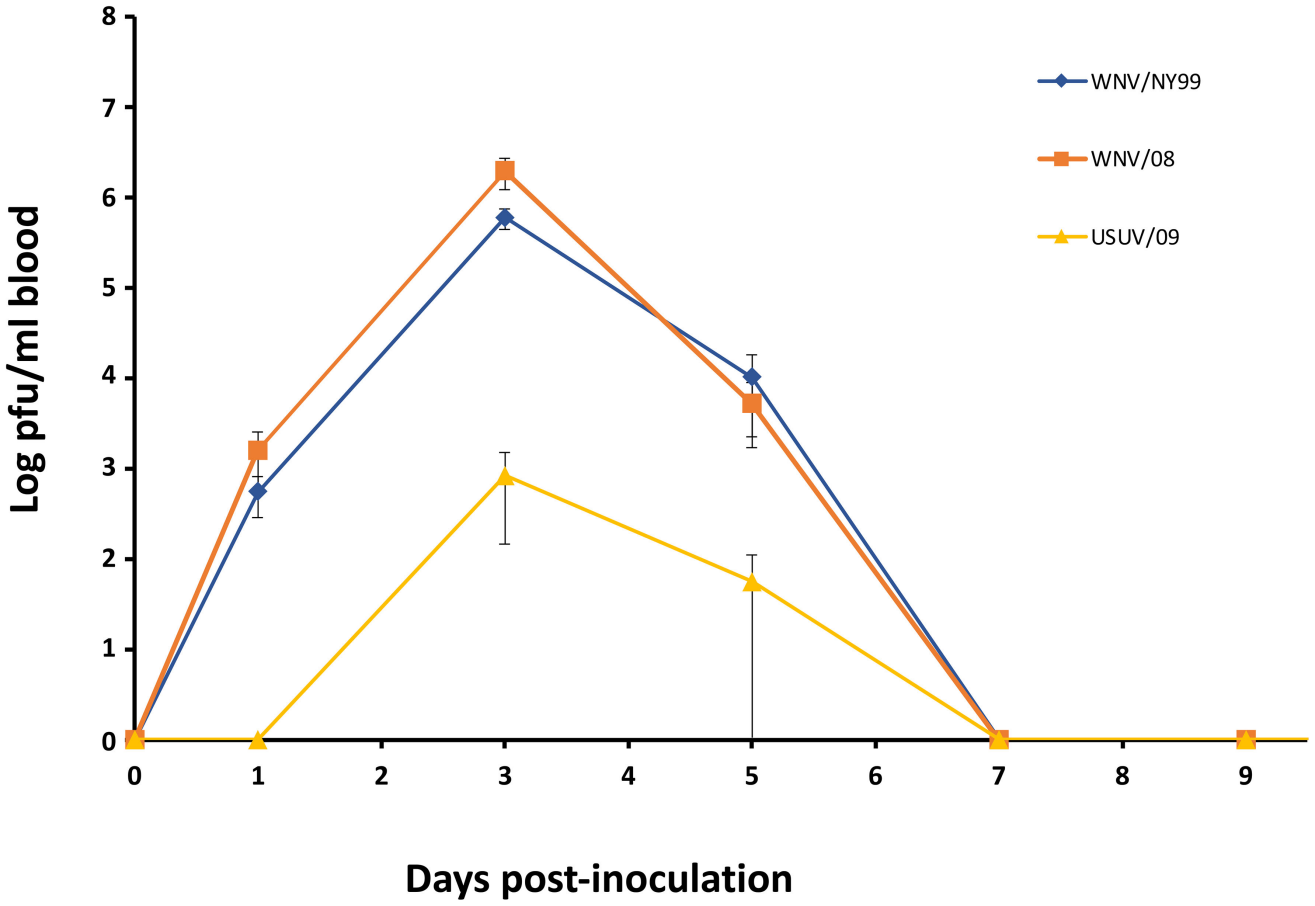
Mean daily viremia for the three inoculated virus groups. Each point represents the mean viremia for the surviving individuals at different times post-inoculation. Bars represent the standard error of the mean.

All inoculated partridges developed detectable viremia by plaque assay for both WNV strains analyzed. The viremia peak was observed at 3 dpi. Statistically significant differences were detected between the two WNV-inoculated groups, with WNV/08 giving higher viremia titers (1.97 × 10^6^ pfu/ml) than WNV/NY99 (6 × 10^5^ pfu/ml) (p = 0.04). Only three partridges developed detectable viremia at 3 dpi for USUV/09 and only one at 5 dpi. The viremia peak was also observed at 3 dpi with a mean of 8 × 10^2^ pfu/ml (**Figure 3**).

**Figure 4:**
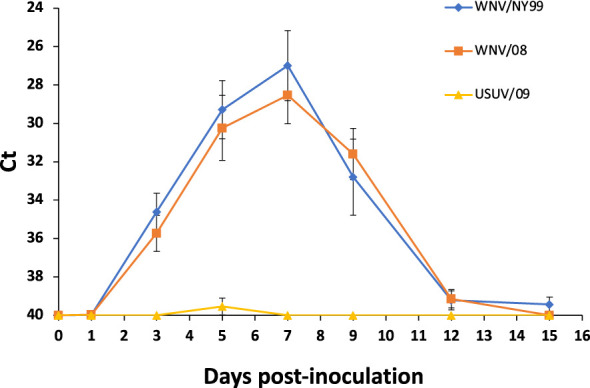
Viral genome load of feather pulps collected from the surviving individuals of the three inoculated virus groups at different days post-inoculation. Bars represent standard error of the mean.

In feathers, viral genome load was similar in WNV/NY99 and WNV/08 groups (χ^2 = ^0.85, d.f. = 1, p-value = 0.36), with the peak at 7 dpi with average Ct values of 26.99 for WNV/NY99 and 28.54 for WNV/08 (**Figure 4**). For USUV/09-infected birds, only one individual showed viral genome in feathers at 5 dpi with a Ct value of 35.86 (**Figure 4**).

**Figure 5:**
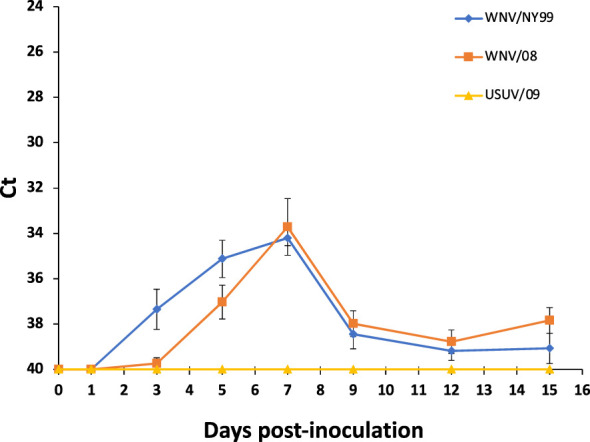
Viral genome load of oropharyngeal swabs collected from the surviving individuals of the three inoculated virus groups at different days post-inoculation. Bars represent standard error of the mean.

West Nile virus genome was detected in oral swabs of the inoculated partridges from 3 dpi to the end of the experiment (15 dpi) but at lower viral RNA loads than in blood and feathers. Statistically significant differences were not observed in the two WNV-inoculated groups (χ^2 = ^0.13, d.f. = 1, p-value = 0.72). The peak of viral RNA load in swabs was reached at 7 dpi for both WNV strains at Ct values of 34.20 for WNV/NY99 and 33.72 for WNV/08 (**Figure 5**). In the USUV-inoculated group, the virus genome was detected in oral swabs only in two birds at 7 dpi at extremely low rates (Ct values of 39.49 and 39.97, respectively).

At the end of the experiment, in the WNV/NY99-inoculated group, low viral RNA loads were detected in some of the organs (brain, heart, kidney, or spleen) of the three animals analyzed. The highest viral RNA load was obtained in the brain, while the virus genome was completely absent from the liver. In the group inoculated with WNV/08, the viral genome was found only in the heart and spleen from one out of three analyzed birds at 15 dpi, with the highest viral RNA load detected in the heart. In the bird that succumbed to the WNV/08 infection at 7 dpi, the viral genome was detected in all the organs analyzed (brain, heart, liver, kidney, and spleen), with the highest viral RNA load in the kidney and the lowest in the liver. The Usutu virus genome was not detected in any of the organs of the three animals analyzed at 15 dpi (**Table 2**).

**Table 2:** Virus genome (Ct values) in organs by day post-inoculation and virus strain.

Virus strain	dpi	Brain	Heart	Kidney	Liver	Spleen
WNV/NY99	15	Neg.	37.9	Neg.	Neg.	Neg.
	15	33.8	Neg.	37.5	Neg.	Neg.
	15	36	Neg.	Neg.	Neg.	37.1
WNV/08	7*	26.6	26.7	24.6	37.9	26.4
	15	Neg.	32.9	Neg.	Neg.	35.2
	15	Neg.	Neg.	Neg.	Neg.	Neg.
	15	Neg.	Neg.	Neg.	Neg.	Neg.
USUV/09	15	Neg.	Neg.	Neg.	Neg.	Neg.
	15	Neg.	Neg.	Neg.	Neg.	Neg.
	15	Neg.	Neg.	Neg.	Neg.	Neg.

Ct values ≥40 are considered negative (neg.).

^*^ Lethally infected partridge.

### Seroconversion

**Figure 6:**
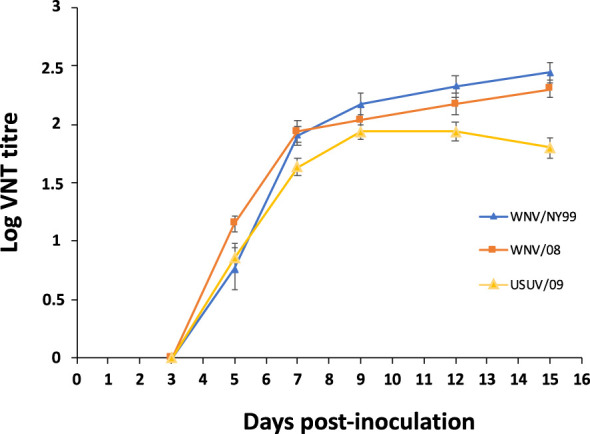
Neutralizing antibody response in serum of inoculated red-legged partridges measured by virus neutralization test. Bars represent standard error of the means.

All surviving partridges inoculated with WNV and USUV developed antibodies. Neutralizing antibodies were first observed at 5 dpi. By day 7, all inoculated individuals had seroconverted, reaching maximum neutralizing antibody titers at 9–15 dpi (**Figure 6**).

## Discussion

Our results show that red-legged partridges are susceptible to WNV/NY99, WNV/08, and USUV/09 infection. However, on the basis of the observed viremia and viral loads in blood, it seems that this species is a competent host for analyzed WNV isolates but not for USUV.

Previous studies have evidenced that red-legged partridges are susceptible to infection and disease and are transmission-competent hosts for several L1 and L2 WNV strains (Sotelo et al., 2011; Pérez-Ramírez et al., 2018). In these studies, important differences in terms of host competence capacity and pathogenicity for the different strains studied were observed (Sotelo et al., 2011; Pérez-Ramírez et al., 2018). However, the WNV strains analyzed in our study (WNV/08 and WNV/NY99) did not differ in either viral loads in blood or pathogenicity for the infected animals. Both strains produced similar morbidity, and although one individual infected with WNV/08 died, this did not mean a significant difference in mortality between both strains. Indeed, viral load profiles in organs, feathers, and oropharyngeal swabs during the infection were also similar. Infection of both WNV strains analyzed (WNV/NY99 and WNV/08) induced viremia values over 10^5^ pfu/ml, which is the value considered as the threshold necessary to infect a mosquito that may feed on an infected bird (see Komar et al., 2003). Consequently, the red-legged partridge would constitute a competent host for these two strains. Thus, our results support a potential role for the red-legged partridge in the transmission of WNV in Southern Spain, where this species is endemic and the virus circulates intensively with new cases reported in every transmission season (Figuerola et al., 2022).

Regarding USUV, the virus has caused numerous deaths in birds in European countries (Weissenböck et al., 2002; Ziegler et al., 2015; Vilibic-Cavlek et al., 2020), and USUV-specific antibodies have been found in a larger range of wild bird species (Figuerola et al., 2009; Manarolla et al., 2010; Becker et al., 2012; Llorente et al., 2013; Michel et al., 2019; Bravo-Barriga et al., 2021; Napp et al., 2021; Marzal et al., 2022), evidencing exposure to USUV infection and its circulation in different European countries. However, little is known about the competence capacity of different bird species for USUV transmission due to the very scarce experimental studies performed in wild birds, particularly in natural reservoir hosts. To our knowledge, USUV experimental infection trials have been carried out in only five avian species: domestic goose (*Anser anser* f. *domestica*) (Chvala et al., 2006), domestic chicken (*Gallus gallus domesticus*) (Chvala et al., 2005; Kuchinsky et al., 2021), domestic canary (*Serinus canaria*) (Benzarti et al., 2020), Eurasian magpie (Escribano-Romero et al., 2021), and house sparrow (Kuchinsky et al., 2022). Only canaries and sparrows developed viremia levels high enough to be considered competent hosts (Benzarti et al., 2020; Kuchinsky et al., 2022). However, domestic geese, Eurasian magpies, and domestic chickens infected with USUV did not show clinical signs and did not develop high viremia levels (Chvala et al., 2005; Chvala et al., 2006; Escribano-Romero et al., 2021).

From the data of viremia and viral load in blood obtained in this study, we have found out that the red-legged partridge is susceptible to USUV infection. Nevertheless, only 30% of the infected animals showed detectable viremia, and the mean peak of viremia at 3 dpi is very low, under 10^3^, being lower than the threshold necessary to infect a mosquito (Kuchinsky et al., 2022). Considering these data, partridge is not a competent host for USUV.

While we did not observe relevant differences in mortality rates between WNV and USUV, important differences were evidenced in terms of morbidity. West Nile virus-infected animals suffered a significant delay in weight gain as compared to the control birds. By contrast, USUV-infected partridges gained weight at a similar rate as the control group, which suggests that USUV is not pathogenic for red-legged partridges. Similar results were found for domestic canaries and house sparrows by Benzarti et al. (2020) and Kuchinsky et al. (2022), respectively.

The USUV/09 strain has been inoculated previously in 2-day-old chickens by Kuchinsky et al. (2021). They found that all the chickens survived and that no clinical signs of disease, including weight loss, were observed, but the individuals showed high viremia levels (i.e., >5 Log_10_ pfu/ml) (Kuchinsky et al., 2021). These viremia values could be due to the age of the individuals considering that 2-week-old chickens did not show high viremia (Chvala et al., 2005). In addition, the USUV/09 strain has been also inoculated in knockout mice (interferon α/β receptor 1 knockout (Ifnar1−/−)) showing similar pathogenicity as the African USUV strains (South Africa 1959, Uganda 2010, and Senegal 2003) and higher pathogenicity than the European USUV strain (USUV Netherlands 2016) (Kuchinsky et al., 2020).

Interestingly, the previous studies of experimental USUV infections in birds (Benzarti et al., 2020; Kuchinsky et al., 2022) point to an association of host competence and susceptibility to USUV infection with bird families, where passerines (sparrow and canary) are competent hosts and highly susceptible to USUV infection, whereas ducks and chickens (Anatidae and Phasianidae families, respectively) seem to be not competent hosts and barely susceptible to USUV infection. This is in agreement with the field observations, where most USUV detections in Europe are found in passerines. Our results observed in red-legged partridges, belonging to the Phasianidae family, are in line with these observations. However, more USUV experimental infection studies with other avian species are needed to find out which species could act as competent hosts or even as super spreaders. Likewise, differences in viremia levels or clinical signs after infection could vary depending on the USUV strain involved, as already demonstrated for WNV. Until now, to our knowledge, only one study has evaluated the susceptibility of wild birds (i.e., house sparrows) to different USUV strains (Uganda 2012 and the Netherlands 2016 belonging to Africa 3 and Europe 3 lineages, respectively), finding differences in the competence between them (Kuchinsky et al., 2022). It would be advisable to experimentally assess other recent WNV and USUV strains circulating in Europe to gain a better understanding of the transmission dynamics of these viruses in the wild.

## Conclusion

In summary, we established that red-legged partridges are susceptible to the infection by WNV/08 strain, showing a similar pathogenicity as for WNV/NY99 strain. However, although red-legged partridges are susceptible to the infection by USUV/09, they are not competent hosts for the transmission of this strain. Further studies using different avian species and a variety of WNV and USUV strains co-circulating in Europe are necessary to understand the complex transmission dynamics of both viruses.

## Data availability statement

The raw data supporting the conclusions of this article will be made available by the authors, without undue reservation.

## Ethics statement

Animal care, handling and experimental procedures were authorized by the INIA Committee of Ethics and Animal Experimentation (Reference: 10/033826).

## Author contributions

FL, MJ-C, and AV conceived and designed the study. FL, EP-R, RG-L, LH, and AV performed the methodology and the experimental infection. FL, EP-R, and RG-L analyzed the samples and the data. RGL led the writing of the manuscript. FL, RG-L, EP-R, LH, MS-S, MJ-C, and AV reviewed and contributed critically to the drafts. All authors contributed to the article and approved the submitted version.
